# Altered Regional Homogeneity in the Development of Minimal Hepatic Encephalopathy: A Resting-State Functional MRI Study

**DOI:** 10.1371/journal.pone.0042016

**Published:** 2012-07-25

**Authors:** Ling Ni, Rongfeng Qi, Long Jiang Zhang, Jianhui Zhong, Gang Zheng, Zhiqiang Zhang, Yuan Zhong, Qiang Xu, Wei Liao, Qing Jiao, Xingjiang Wu, Xinxin Fan, Guang Ming Lu

**Affiliations:** 1 Department of Medical Imaging, Jinling Hospital, Clinical School of Medical College, Nanjing University, Nanjing, China; 2 Department of Biomedical Engineering, Zhejiang University, Hangzhou, Zhejiang, China; 3 Key Laboratory for NeuroInformation of Ministry of Education, School of Life Science and Technology, University of Electronic Science and Technology of China, Chengdu, China; 4 Department of General Surgery, Jinling Hospital, Clinical School of Medical College, Nanjing University, Nanjing, China; Hangzhou Normal University, China

## Abstract

**Background:**

Little is known about how spontaneous brain activity progresses from non-hepatic encephalopathy (non-HE) to minimal HE (MHE). The purpose of this study was to evaluate the evolution pattern of spontaneous brain activities in cirrhotic patients using resting-state fMRI with a regional homogeneity (ReHo) method.

**Methodology/Principal Findings:**

Resting-state fMRI data were acquired in 47 cirrhotic patients (minimal HE [MHE], n = 20, and non-HE, n = 27) and 25 age-and sex-matched healthy controls. The Kendall’s coefficient of concordance (KCC) was used to measure the regional homogeneity. The regional homogeneity maps were compared with ANOVA tests among MHE, non-HE, and healthy control groups and *t*-tests between each pair in a voxel-wise way. Correlation analyses were performed to explore the relationships between regional ReHo values and Child-Pugh scores, number connection test type A (NCT-A), digit symbol test (DST) scores, venous blood ammonia levels. Compared with healthy controls, both MHE and non-HE patients showed decreased ReHo in the bilateral frontal, parietal and temporal lobes and increased ReHo in the bilateral caudate. Compared with the non-HE, MHE patients showed decreased ReHo in the bilateral precuneus, cuneus and supplementary motor area (SMA). The NCT-A of cirrhotic patients negatively correlated with ReHo values in the precuneus, cuneus and lingual gyrus. DST scores positively correlated with ReHo values in the cuneus, precuneus and lingual gyrus, and negatively correlated with ReHo values in the bilateral caudate (*P*<0.05, AlphaSim corrected).

**Conclusions/Significance:**

Diffused abnormal homogeneity of baseline brain activity was nonspecific for MHE, and only the progressively decreased ReHo in the SMA and the cuneus, especially for the latter, might be associated with the development of MHE. The ReHo analysis may be potentially valuable for detecting the development from non-HE to MHE.

## Introduction

Minimal hepatic encephalopathy (MHE) refers to a subgroup of cirrhotic patients without clinical overt hepatic encephalopathy (OHE) symptoms but with abnormalities in brain metabolism, motor and neuro-cognitive functions [1], [2]. MHE can have a far-reaching impact on quality of life [3] and a high likelihood of development to OHE [4]. In particular, MHE, with the prevalence of 30–80% in cirrhotic patients, may affect the ability of driving that requires constant vigilance and coordination [5] and may be a significant factor behind motor vehicle accidents. Therefore, the early detection of MHE is crucial for the prompt treatment of these patients and the improvement of their prognosis [6].

Recently, blood oxygenation level-dependent [7] functional magnetic resonance imaging (BOLD-fMRI) has been increasingly applied for investigating the neuro-pathophysiological mechanism of HE. Several task-related [8], [9], [10] and resting-state fMRI (rs-fMRI) [11], [12] studies have demonstrated functional impairments in MHE or OHE patients and negative correlations of these brain abnormalities with clinical marks of HE, such as venous blood ammonia levels. Of fMRI analysis methods, regional homogeneity (ReHo) developed by Zang et al. [13], has been widely used to investigate the functional modulations in many brain diseases such as major depression [14], Parkinson’s Disease [15], and Alzheimer’s disease [16]. ReHo assumes that within a functional cluster, the hemodynamic characteristics of every voxel would be similar or synchronous with that of others, and such similarity could be changed or modulated by different conditions [13]. More recently, Chen et al. [17] used ReHo to investigate the resting brain activity in MHE patients, and found abnormal local neuronal activity in MHE. However, whether these abnormal local neuronal activities in MHE patients are specific for MHE remains unclear. Although the above-mentioned functional neuroimaging methods have been used to investigate HE [11], [12], the progression patterns from non-hepatic encephalopathy (non-HE) to MHE have been less explored and the exact neuro-pathophysiological progression mechanism from non-HE to MHE remains unknown.

Our hypothesis is that there is one or several key brain regions abnormally triggered during the development of MHE. To valid this hypothesis, we used ReHo algorithm in the current rs-fMRI study to evaluate the progressive patterns of regional spontaneous activities from non-HE to MHE throughout the whole brain.

**Table 1:** Demographics and clinical data of cirrhotic patients and healthy controls.

Protocols	Controls (n = 25)	Non-HE (n = 27)	MHE (n = 20)	*P* value
Sex (M/F)	13/12	20/7	13/7	0.252^a^
Age (±SD), y	55±8(42–64)	51±6 (41–64)	55±7 (42–67)	0.108^b^
DST (score)	44.68±8.28 (26–66)	40.11±8.80 (30–66)	23.15±8.17 (9–37)	<0.01^c^
NCT-A (s)	46.32±9.09 (31–64)	45.78±8.53 (21–60)	72.80±16.71 (67–140)	<0.01^c^
Venous ammonia (in µmol/L)	–	51.10±33.54 (9–113)	69.06±26.13 (9–109)	0.072^d^
Child-Pugh scale				
A	–	19	12	
B	–	6	8	
C	–	2	0	

Values are expressed as mean ±SD. MHE = minimal hepatic encephalopathy; DST = digital symbol test; NCT-A = number connection test-A; Non-HE = non-hepatic encephalopathy.

a The *P* value for gender distribution in the three groups was obtained by chi-square test.

b The *P* value for age distribution in the three groups was obtained by one-way analysis of variance test.

c The *P* value for difference of the neuropsychological test scores among the three groups was obtained by one-way analysis of variance test.

d The *P* value for difference of venous blood ammonia between the two groups was obtained by two-sample *t* test.

## Material and Methods

### Subjects

The study was approved by the Medical Research Ethics Committee of Jinling Hospital, Nanjing, China, and all the subjects’ written informed consents were obtained before the study. The patients were recruited from patients hospitalized at Jinling Hospital, Nanjing, China. Forty seven cirrhotic patients were included for this study. Chronic cirrhotic patients without OHE were included. The following exclusion criteria were applied in this study: (a) overt HE (episodic or persistent) as revealed by a standard clinical neurological investigation, (b) any drug/alcohol abuse history, (c) any brain lesions such as tumor, stroke assessed on basis of medical history, (d) known psychiatric disorders, (e) head motion more than 1.0 mm or 1.0° during MR scanning.

**Figure 1:**
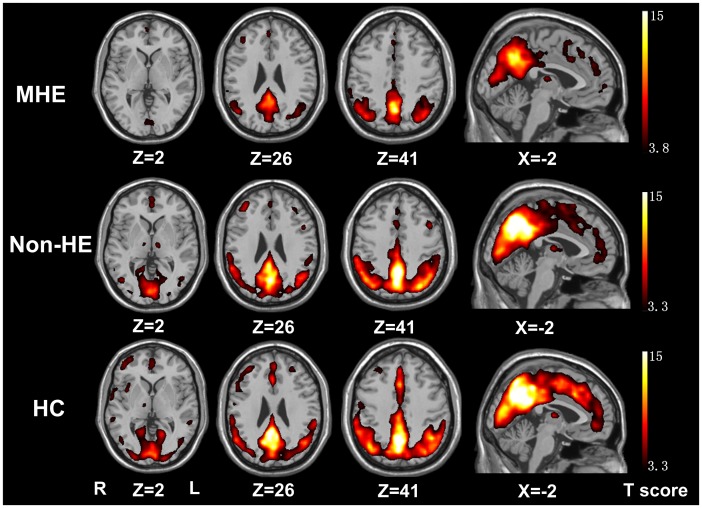
Mean ReHo maps within the MHE, non-HE and healthy control groups (*P*<0.01, FDR corrected). The PCC/precuneus, MPFC and bilateral IPL exhibit significantly higher ReHo values than the global mean ReHo value within each group, but with a different strength among the groups. MHE = minimal hepatic encephalopathy; non-HE = non-hepatic encephalopathy; ReHo = regional homogeneity; FDR = false discovery rate; PCC = posterior cingulate cortex; MPFC = medial prefrontal cortex; IPL = inferior parietal lobe.

MHE was defined using neuropsychological tests, including the number connection test-A (NCT-A) and digit-symbol test (DST) recommended by Weissenborn et al. [2]. MHE was diagnosed when the scores of at least one test were beyond 2SD (standard deviation) of the mean value for the age-matched controls. According to neuropsychological tests, 20 patients were diagnosed as MHE, and 27 patients as no HE (non-HE).

**Figure 2:**
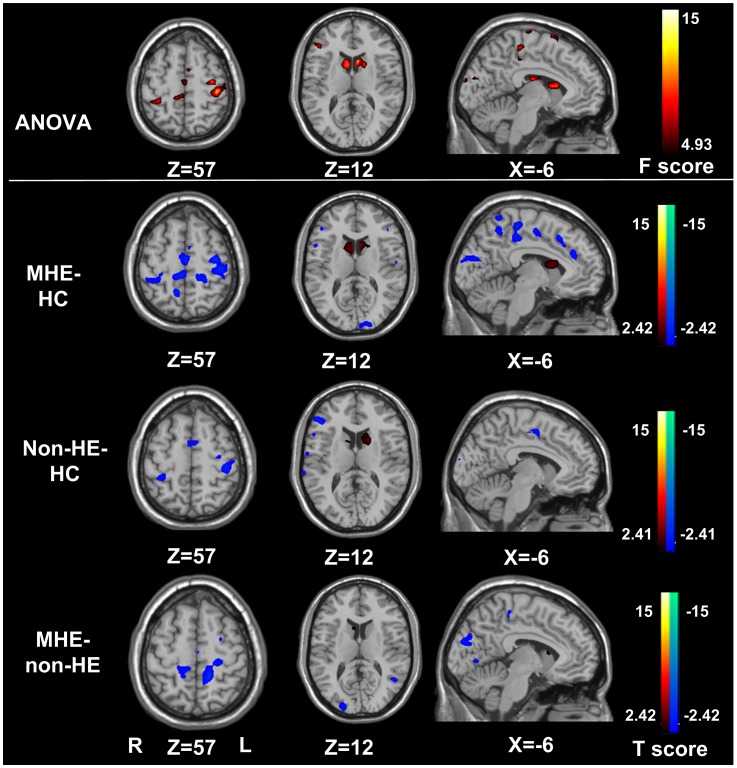
ReHo differences among non-HE, MHE, and healthy controls (*P*<0.05, AlphaSim corrected). Compared with the healthy controls, MHE patients show significantly decreased ReHo in the bilateral frontal lobes including the left ACC, parietal lobes including the PCu, temporal lobes, occipital lobes including the cuneus and increased ReHo in the bilateral caudate, and non-HE patients show decreased ReHo in the bilateral frontal lobes, parietal lobes, temporal lobes and increased ReHo in the bilateral caudate. Compared with the non-HE patients, the MHE patients show decreased ReHo in the bilateral parietal lobes including the PCu, SMA, frontal lobes and occipital lobes including the cuneus. MHE = minimal hepatic encephalopathy; non-HE = non-hepatic encephalopathy; HC = healthy controls; ReHo = regional homogeneity; PCu = precuneus; ACC = anterior cingulate cortex; SMA = supplementary motor area.

All patients had complete laboratory tests to evaluate liver function and venous blood ammonia values before MR examination. The liver dysfunction was determined according to the Child-Pugh score [18], which graded Child-Pugh A in 31 patients, Child-Pugh B in 14 patients, and Child-Pugh C in 2 patients.

Twenty five age-and sex-matched healthy controls were recruited from the local community by advertisements. All the healthy controls had no disease of liver and other systems, or any history of psychiatric or neurological diseases. All subjects were self-identified as right-handed with normal sight.

**Table 2:** Regions showing ReHo differences between MHE patients and healthy controls.

Brain regions	BA	MNI coordinates (mm)	Vol (mm^3^)	Maximal T value
		(x, y, z)		
Left PreCG	11/31	−51,−18,42	3807	−2.42
Left PoCG	40	−24,−30,57	6345	−2.42
Right SMA	32	6,−6,54	783	−2.42
Right STG	12	60,−15,3	729	−2.42
Right PreCG	4/6	24,−24,66	459	−2.42
Left cuneus	18	−6,−96,18	567	−2.43
Right MFG	24	−9,3,57	1890	−2.43
Right IPL	4/6	30,−33,54	1863	−2.43
Right PoCG	40/3	36,−42,63	2916	−2.43
Right cuneus	19	3,−87,24	567	−2.44
Left SMA	6	−9,3,54	1701	−2.44
Right ACC	32	6,36,21	621	−2.45
Left IPL	40	−42,−36,15	1458	−2.46
Left STG	14	−54,−45,27	432	−2.46
Left ACC	32	−9.29,−10	272	−2.50
Right PCu	7	9,−54,48	1107	−2.51
Left MFG	24	−3,0,42	459	−2.51
Left PCu	7	−15,−78,42	459	−2.57
Right caudate		9,9,12	837	+4.54
Left caudate		−9,15,9	1917	+4.74

Positive sign represents increase, and negative sign represents decrease. ReHo = regional homogeneity; MHE = minimal hepatic encephalopathy; MNI = Montreal Neurological Institute; IPL = inferior parietal lobule; PreCG = precentral gyrus; PoCG = postcentral gyrus; PCu = precuneus; SMA = supplementary motor area; MFG = medial frontal gyrus; STG = superior temporal gyrus; ACC = anterior cingulate cortex; BA = brodmann area. *P*<0.05, Alphasim corrected.

**Table 3:** Regions showing ReHo differences between non-HE patients and healthy controls.

Brain regions	BA	MNI coordinates (mm)	Vol (mm^3^)	Maximal T value
		(x, y, z)		
Right IFG	9	42,3,36	1323	−2.41
Right SMA	32	6,−6,54	675	−2.41
Left MFG	24	−3,0,42	1080	−2.41
Left SMA	6/24	−6,0,57	1134	−2.42
Left PreCG	4/2	−36,−24,60	1215	−2.43
Left PoCG	3	−39,−33,63	2268	−2.45
Right STG	12	60,−15,3	1674	−2.45
Right caudate		15,−6,18	675	+4.50
Left caudate		−9,6,15	1836	+5.09

Positive sign represents increase, and negative sign represents decrease. ReHo = regional homogeneity; MNI = Montreal Neurological Institute; PoCG = postcentral gyrus; PreCG = precentral gyrus; IFG = inferior frontal gyrus; STG = superior temporal gyrus; SMA = supplementary motor area; MFG = medial frontal gyrus; BA = brodmann area. *P*<0.05, Alphasim corrected.

**Table 4:** Regions showing ReHo differences between MHE and non-HE patients.

Brain regions	BA	MNI coordinates (mm)	Vol (mm^3^)	Maximal T value
		(x, y, z)		
Left PCu	7	−15,−78,42	1620	−2.42
Left PreCG	3/5	−9,−24,−12	648	−2.42
Left cuneus	7/19	−9,−87,21	2106	−2.42
Right PCu	31	21,−69,30	2619	−2.42
Right cuneus	18	9,−35,21	2538	−2.42
Left PoCG	2/4	−15,−30,66	1269	−2.43
Right SMA	6/24	6,0,57	459	−2.52

Positive sign represents increase, and negative sign represents decrease. ReHo = regional homogeneity; MHE = minimal HE; MNI = Montreal Neurological Institute; PCu = precuneus; PreCG = precentral gyrus; PoCG = postcentral gyrus; SMA = supplementary motor area; BA = brodmann area. *P*<0.05, Alphasim corrected.

### MRI Data Acquisition

Imaging data were acquired on a 3 Tesla MR scanner (TIM Trio, Siemens Medical Solutions, Erlangen, Germany). All subjects were placed in a standard head coil and fitted to foam padding to reduce head motion. They were instructed to hold still, keep eyes closed but be awake in the MR scanner. High-resolution axial T_1_- FLASH sequence images were obtained in every subject to detect clinically silent lesions (30 axial sections; section thickness = 4 mm; intersection gap = 0.4 mm; in-plane resolution = 320×256; field of view (FOV) = 240×240 mm^2^; repetition time (TR) = 350 ms; echo time (TE) = 2.46 ms). A gradient-echo echo-planar (GRE-EPI) sequence sensitive to BOLD contrast was used to acquire functional images (TR = 2000 ms, TE = 30 ms, ﬂip angle = 90°, FOV = 240×240 mm^2^, matrix = 64×64, slice thickness = 4 mm and slice gap = 0.4 mm). Each brain volume comprised 30 axial slices and each functional run contained 250 volumes. The sections were approximately along the anterior commissure–posterior commissure line and covered about −30 to 60 mm in the inferior-superior direction. Each fMRI scan lasted 500 s.

**Figure 3:**
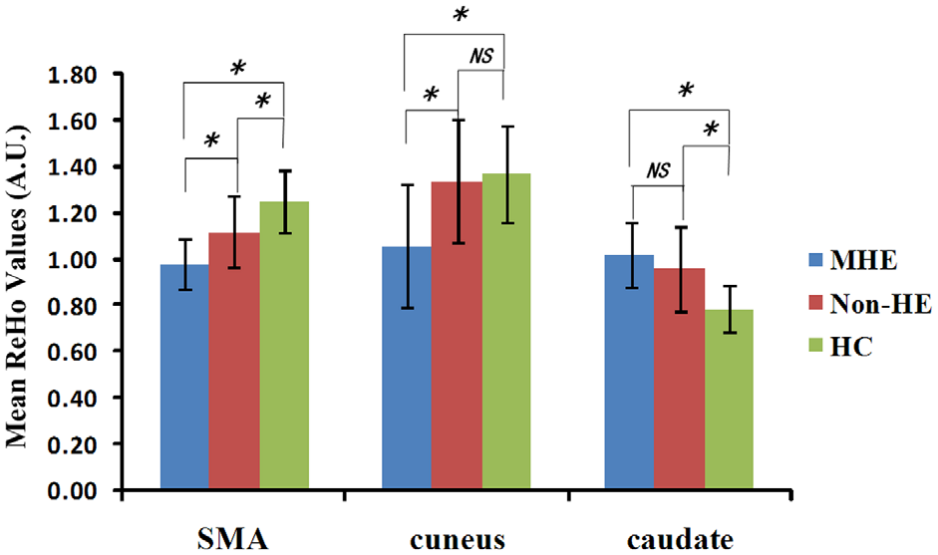
The mean ReHo values of the cuneus, SMA, caudate among the three groups. The mean ReHo values of the cuneus show a decreasing trend of ReHo_controls_ (1.37±0.25) > ReHo_non-HE_ (1.34±0.23) > ReHo_MHE_ (1.08±0.24) (*F = *8.263, *P = *0.001). The mean ReHo values of the caudate show an increasing trend of ReHo_controls_ (0.81±0.06) < ReHo_non-HE_ (0.96±0.08) < ReHo_MHE_ (1.03±0.17) (*F = *25.317, *P*<0.001). The mean ReHo values of the SMA progressively decrease from controls (1.22±0.12) to non-HE (1.11±0.13) to MHE (1.01±0.15) (all *P*<0.05, Bonferroni corrected). ReHo  =  regional homogeneity; A.U. = arbitrary unit; SMA = supplementary motor area; MHE = minimal hepatic encephalopathy; Non-HE = non-hepatic encephalopathy; HC = healthy control; *NS* = no significance. *represents for *P<*0.05 (Bonferroni corrected).

### Image Preprocessing

Image preprocessing was conducted using Statistical Parametric Mapping software (SPM8, http://www.fil.ion.ucl.ac.uk/spm/). The first ten volumes of the functional images were discarded for the signal equilibrium and participants’ adaptation to the scanning circumstance. The remaining 240 time points were left for further analysis. The slice timing, head motion correction and spatial normalization to the standard Montreal Neurological Institute (MNI) template with a resampled voxel size of 3×3×3 mm^3^ were conducted. No participant had head motion of more than 1.0 mm maximum translation in any of the x, y or z directions or 1.0 degree of maximum rotation about three axes during scanning. We also evaluated the group differences in translation and rotation of head motion according to the formula 1 [19]:
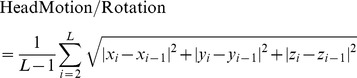
(1)where *L* is the length of the time series (*L = *240 in this study), x*i*, y*i* and z*i* are translations/rotations at the *i*th time point in the x, y and z directions, respectively. The results showed that the three groups had no significant differences (one - way analysis of variance, *F* = 0.252, *P = *0.778 for translational motion and *F* = 0.399, *P = *0.672 for rotational motion). Resting State fMRI Data Analysis Toolkit (REST) Version 1.5 (http://www.restfmri.net) was then used for removing the linear trend of time courses and for temporally band-pass filtering (0.01–0.08 Hz) [20]–[21] to reduce low-frequency drift and physiological high frequency respiratory and cardiac noise.

**Figure 4:**
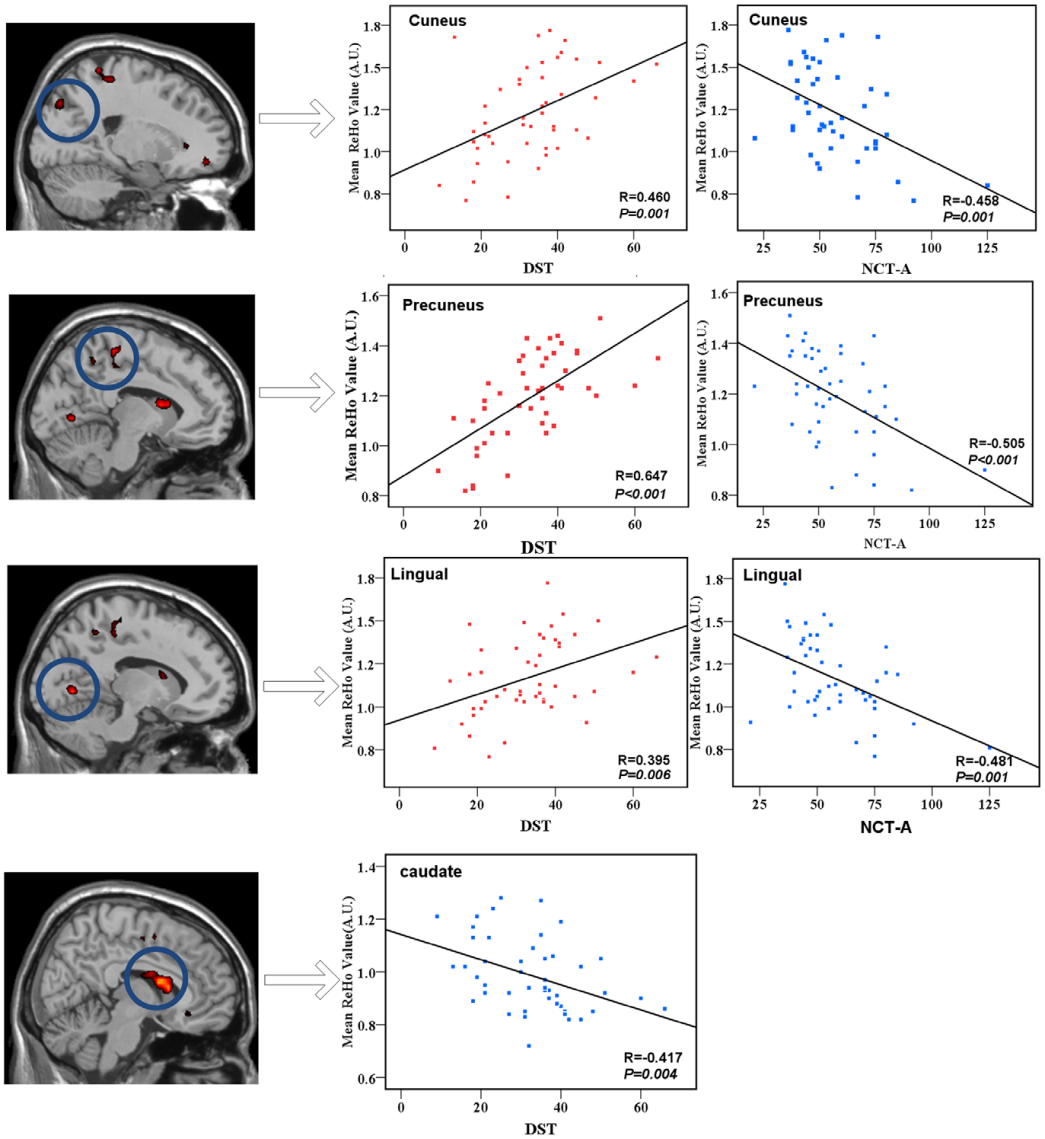
The correlation between the ReHo values and DST scores, as well as NCT-A in the combined MHE and non-HE groups (*P*<0.05, AlphaSim corrected). The DST scores positively correlate with ReHo values in the bilateral occipital lobes including cuneus and lingual gyrus, parietal lobes including the PCu (warm color), and negatively correlate with ReHo values in the bilateral caudate (cold color). The NCT-A has a negative correlation with the ReHo values in the bilateral parietal lobes including the PCu, occipital lobes including the cuneus and lingual gyrus. DST = digital symbol test; NCT-A = number connection test type A; ReHo = regional homogeneity.

### ReHo Analysis

The ReHo analysis was performed for each subject by the REST software. A Kendall’s coefficient of concordance (KCC) value (also called ReHo value) was calculated to measure the similarity of the ranked time series of a given voxel to its nearest 26 neighbor voxels in a voxel-wise way with the formula 2:
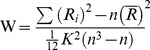
(2)where W is the KCC for a given voxel, ranging from 0 to 1; R*i* is the sum rank of the *i*th time point; 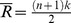 is the mean of the R*i*s; k is the number of time series within a measured cluster (27, one given voxel plus the number of its neighbors); n is the number of ranks (here, n = 240 time points).

Through calculating the KCC value of every voxel in the whole brain, an individual ReHo map was obtained for each subject. The intracranial voxels were extracted to make a mask [22]. For standardization purposes, each individual ReHo map was divided by its own mean ReHo within the mask. Spatial smoothing was then performed with a 4-mm full-width at half-maximum (FWHM) Gaussian kernel.

### Statistics Analysis

Statistical analysis was performed using the software SPSS version 16.0 (SPSS Inc. Chicago, IL) for demographic and clinical data, and SPM8 (statistical parametric mapping, http://www.fil.ion.ucl.ac.uk/spm/) for fMRI data. A second-level random-effect one-sample *t* test was performed to show the ReHo results for each group (MHE, non-HE and healthy control), the threshold was set at *P*<0.01, corrected with false discovery rate (FDR) criterion. To explore the ReHo differences among the three groups (MHE, non-HE patients, and healthy controls), a one-way analysis of variance (ANOVA) was performed on the individual normalized ReHo maps in a voxel-by-voxel manner. Age, gender and head motion were included as covariates in the present and following functional data statistic analysis. The result was corrected using the Alphasim program, which setting at *P*<0.01 and cluster size >189 mm^3^, which corresponded to a corrected *P*<0.05. If statistical difference was present, post hoc *t*-tests were performed to detect the inter-group difference of brain regions. In order to constrain the results to the clusters identified in the one-way ANOVA, we made a mask including regions showing significant group differences in the aforementioned ANOVA analysis, and restricted the post hoc analyses results within this mask. With this mask, the results of each pair were set at *P*<0.01 and cluster size >189 mm^3^, which corresponded to a corrected *P*<0.05. The first three regions showing the most significantly altered ReHo in ANOVA were selected as seed regions, then ReHo values were extracted from these seed regions within each subject and compared among three groups. Comparative analysis of these quantitative ReHo values was performed using SPSS 16.0 (SPSS Inc., Chicago, IL), significant level of *P* values were set at less than 0.05, corrected for multiple comparison using the Bonferroni correction.

Finally, the regions showing significant among-group differences were extracted as regions of interest (ROIs), then Pearson correlation analysis was performed to measure the relationship between the mean ReHo values of these ROIs of each patient and Child-Pugh scores, NCT-A/DST scores, venous blood ammonia values, *P*<0.05 (AlphaSim corrected) was used to determine the significant correlation.

## Results

Demographics and clinical data for cirrhotic patients and healthy subjects were summarized in Table 1. There were no significant differences in age (*P* = 0.108) and gender (*P* = 0.252) among the three groups. The NCT-A and DST scores among the three groups showed significant difference (both *P*<0.01). The difference of venous blood ammonia values between the two patient group didn’t reach the statistical significance (*P = *0.072).

ReHo results within each group were shown in Fig. 1 (*P*<0.01, FDR correction). The posterior cingulate cortex (PCC)/precuneus, medial prefrontal cortex (MPFC), and bilateral inferior parietal lobe (IPL) exhibited significantly higher ReHo values than the global mean ReHo value.

Compared with the healthy control group, both MHE and non-HE patients showed decreased regional homogeneity in the bilateral frontal, parietal and temporal lobes and increased regional homogeneity in the bilateral caudate (Fig. 2, Tables 2 and 3). The regional homogeneity in the bilateral precuneus, cuneus in MHE was also lower than that of the healthy control (Fig. 2, Table 2). In addition, when comparing with the non-HE patients, MHE patients showed decreased regional homogeneity in the bilateral precuneus, cuneus, SMA and medial frontal cortex (MFC) (*P*<0.05, AlphaSim corrected) (Fig. 2, Table 4).

Of the selected 3 seed regions for the quantitative measurements, the ReHo values of the cuneus showed a decreasing trend from ReHo_controls_ (1.37±0.25) to ReHo_non-HE_ (1.34±0.23) to ReHo_MHE_ (1.08±0.24) (*F* = 8.263, *P = *0.001). ReHo values of the cuneus were lower in MHE patients than those of controls (*P = *0.001) and non-HE patients (*P*<0.001), but difference of ReHo values between non-HE patients and controls didn’t reach the level of statistical significance (*P = *0.765) (Fig. 3). The ReHo values of the caudate showed an increasing trend from ReHo_controls_ (0.81±0.06) to ReHo_non-HE_ (0.96±0.08) to ReHo_MHE_ (1.03±0.17) (*F* = 25.317, *P*<0.001). ReHo values of the caudate in the MHE patients and non-HE patients were higher than those of controls (both *P*<0.001), but difference between non-HE and MHE patients didn’t reach the level of statistical significance (*P = *0.124) (Fig. 3). The ReHo values of SMA progressively decreased from controls (1.22±0.12) to non-HE (1.11±0.13) to MHE (1.01±0.15) (all *P*<0.05, Bonferroni corrected) (Fig. 3).

The NCT-A results of all cirrhotic patients negatively correlated with ReHo values in the bilateral precuneus, cuneus and lingual gyrus. DST scores positively correlated with ReHo values in the bilateral cuneus, precuneus and lingual gyrus, and negatively correlated with ReHo values in the bilateral caudate (*P*<0.05, AlphaSim corrected) (Fig. 4). No significant correlations were found between any regional ReHo values and the Child-Pugh scores or venous blood ammonia values in cirrhotic patients.

## Discussion

In this study, we found diffused decreased ReHo values in the cortical regions, and increased ReHo in the bilateral caudate in both MHE and non-HE patients, indicating these changes are not specific for MHE. Patients with MHE showed more widely spread decreased ReHo values in some brain cortices than non-HE patients, suggesting further impairment with the development from non-HE to MHE. These ReHo values in the most identified brain regions correlated with neuropsychological impairments in cirrhotic patients.

Several analysis algorithms have been developed to assess the synchrony of brain activity at rest. For example, seed correlation analysis [24] with hypothesis-driven algorithm has been used to define various specific brain networks. Independent component analysis (ICA) measures the integrated coherence within the entire network. Compared with other resting-state fMRI methods, the ReHo algorithm has the advantage of directly measuring the regional signal similarities across the whole brain [13]. Zang et al. [13] found that in healthy subjects the pattern of resting-state brain activities obtained by the ReHo method was very similar to that observed by positron emission tomography (PET). Although the exact biological mechanism of ReHo remains unclear, some authors suggested that it might reveal the spontaneous brain activity and help improve our understanding of the neuropathological mechanisms underlying many neuropsychological diseases [14], [15].

In our present study, the cirrhotic patients without MHE showed decreased ReHo values mainly in the bilateral parietal lobe including SMA, frontal lobes and temporal lobes, suggesting that the non-HE patients had already potential brain dysfunctions, although they had normal neuropsychological tests similar to normal controls. We also found increased ReHo values in the bilateral caudate in cirrhotic patients. The caudate is crucial in the control of sensory information [23] including the analysis, regulation and modulation of sensory processing. A previous study by Lockwood et al. [26] reported an increased cerebral blood flow in caudate in MHE patients, which was interpreted as a compensatory mechanism for reduced cortical activity detected in that PET study. The similar compensatory mechanism was also reported in many other previous PET studies [26], [27], [28] which showed a redistribution of blood flow and metabolism from various cortical regions to subcortical grey matter regions including caudate. So we speculated that the increased Reho in the present study may also play a compensatory way to the reduced cortical Reho. Thus, resting state fMRI with the ReHo algorithm might potentially detect the brain dysfunction at the early stage of HE (MHE), even in the cirrhotic patients without MHE and OHE. Our current study also indicated most abnormal brain regions measured with the ReHo algorithm were not specific for MHE.

One important finding in our study was that the SMA and cuneus showed progressively decreased ReHo, while the caudate displayed progressively increased ReHo during the development of MHE. Our findings were inconsistent with those in one recently published study by Chen et al [17]. They didn’t report abnormal ReHo values in the SMA and caudate in MHE patients. The exact causes for these differences were unknown, but a possible explanation may be the different field strength MR scanners used (3T in our study vs 1.5T in Chen et al.’s study) and patients with heterogeneous etiology of cirrhosis and different stages of liver dysfunction recruited in these two studies. The SMA had progressively decreased ReHo value from controls to non-HE to MHE, which might be a sensitive mark for evaluating the disease progression but unspecific for the presence of MHE. The SMA plays an important role in planning and initiation of movements [29], [30], thus the decreased synchronization of the SMA may account for the motor dysfunction, especially impaired self-initiated movements, in MHE/HE patients reported in many previous studies [31], [32]. The patients in our present study didn’t show any motor disorder, so our findings suggested that MHE already have abnormal brain function in motor-related regions although without manifested clinical symptoms. The cuneus is considered to be centers of visual processing and inhibitory control [33]. Defects in visual processing and executive functions such as response inhibition in patients with MHE have been described in many previous studies [2], [34]. In this study, we found that regional coherence or synchronization in the bilateral cuneus was decreased in MHE patients while not in non-HE patients, indicating the cuneus might be a specific marker for MHE. This finding is partially supported by the positive correlation between the ReHo values in the cuneus and the DST scores, which also reported in previous rs-fMRI study [17].

Our study had some limitations. First, this study is preliminary and our results are limited to a small sample size with heterogeneous patient etiology, which may affect the statistical analysis and comprehensive interpretation of the results. Further studies with more patients with homogeneous etiology are needed. Second, as a cross-sectional study, we can only observe the progression from non-HE to MHE in different subjects but not in the same patient group recruited in a longitudinal study. A truly longitudinal study is warranted to confirm the finding of this study. Third, motor ability of these patients in our study was not evaluated because these findings were unexpected. Fourth, the exact relationship between the decreased regional ReHo and movement dysfunction remains to be further clarified in the future studies.

In conclusion, diffused abnormal homogeneity of baseline brain activity was observed in all cirrhotic patients, indicating these abnormalities were nonspecific for MHE. The progressively decreased ReHo in the SMA and cuneus, especially for the latter, might be associated with the development of MHE. The ReHo analysis may be potentially valuable for detecting the development from non-HE to MHE.
